# Predictors of and outcomes following orthopaedic joint surgery in patients with early rheumatoid arthritis followed for 20 years

**DOI:** 10.1093/rheumatology/kex172

**Published:** 2017-05-16

**Authors:** James M. Gwinnutt, Deborah P. M. Symmons, Alexander J. MacGregor, Jacqueline R. Chipping, Chloe Lapraik, Tarnya Marshall, Mark Lunt, Suzanne M. M. Verstappen

**Affiliations:** 1 Arthritis Research UK Centre for Epidemiology, Centre for Musculoskeletal Research, School of Biological Sciences, Manchester Academic Health Science Centre, University of Manchester; 2 NIHR Manchester Musculoskeletal Biomedical Research Unit, Central Manchester University Hospitals NHS Foundation Trust, Manchester Academic Health Science Centre, Manchester; 3 Rheumatology Department, Norfolk and Norwich University Hospitals NHS Foundation Trust; 4 Norwich Medical School, University of East Anglia, Norwich, UK

**Keywords:** early rheumatoid arthritis, epidemiology, orthopaedic surgery, functional disability

## Abstract

**Objectives:**

To analyse predictors and outcomes of major orthopaedic surgery in a cohort of RA patients followed for 20 years.

**Methods:**

Patients were recruited to the Norfolk Arthritis Register from 1990 to 1994. Demographic and clinical variables (including the HAQ and swollen and tender joint counts) were assessed at baseline; the 2010 ACR/EULAR RA classification criteria were applied. Patients reported incident comorbidities and major orthopaedic joint surgery (replacement, synovectomy, fusion, excision) when reassessed at years 1, 2, 3, 5, 7, 10, 15 and 20. Baseline and time-varying predictors of orthopaedic surgery were assessed using a conditional risk set model, a type of multiple-failure survival analysis. Change in disability after surgery was assessed using weighted mixed-effects linear regression.

**Results:**

Of 589 RA patients [median age 56 years (IQR 45–68); 66.7% women] recruited to the Norfolk Arthritis Register with at least one follow-up, 102 reported a total of 180 major surgeries, with hip replacement being the most common (*n* = 68/180). Patients reporting major surgery had worse functional disability at all time points, but similar swollen/tender joint counts to those without major surgery. Each unit increase in HAQ score was associated with a doubling of the patient’s risk of having surgery by the next assessment [hazard ratio 2.11 per unit increase in HAQ (95% CI 1.64, 2.71)]. Patients had worse HAQ scores after surgery than patients not undergoing surgery [β = 0.17 (95% CI 0.03, 0.32)].

**Conclusion:**

HAQ was the strongest predictor of future major surgery. This supports the argument that HAQ should be included in routine clinical assessment.

Rheumatology key messages

- The Health Assessment Questionnaire was the strongest independent predictor of future orthopaedic joint surgery.
- Swelling within knee joints strongly predicted knee surgery, suggesting potential benefits of targeted therapy.
- Joint surgery did not improve the overall function of patients; early disease control is essential.

## Introduction

The joint inflammation associated with RA, especially if persistent, may lead to progressive joint damage [1]. Joint damage and pain are major indications for orthopaedic surgery [2, 3]. Over the past two decades the incidence of orthopaedic surgery in patients with RA has been declining, potentially due to better long-term outcomes as a consequence of improvements in treatment [4–6]. For instance, patients with RA had a 12% lower risk of undergoing a total knee replacement between 2003 and 2007 compared with 1993–1997 [7]. However, in a UK study of RA patients from 1986 to 2011, only hand and foot orthopaedic surgery rates declined, with large-joint replacement rates remaining constant over time [8].

Despite this, a significant proportion of RA patients still require orthopaedic surgery [9], and little research on predictors of surgery has been conducted. Kapetanovic *et al.* [10] followed 183 patients with recent-onset RA in Sweden for a mean of 16 years and showed that baseline functional disability (HAQ) [hazard ratio (HR) 1.74/1 unit increase in HAQ (95% CI 1.04, 2.93)], baseline CRP [HR 1.39/10 mg/l increase in CRP (95% CI 1.23, 1.46)] and radiological damage at year 1 [HR 1.47/10 unit increase in Larsen score (95% CI 1.22, 1.77)] were associated with an increased risk of first joint replacement surgery. Moura *et al.* [11], using Canadian physician billing and hospitalization databases with a median 4.6 years follow-up, showed that increased methotrexate exposure within the first year of RA diagnosis was associated with a reduced risk of joint replacement compared with no methotrexate exposure [HR 0.95/month exposure in first year (95% CI 0.93, 0.97)]. An analysis of patients recruited from 1986 to 2012 to the Early RA Study/Early RA Network (ERAS/ERAN) found that high 28-joint DAS (DAS28 >5.1) between years 1 and 5 was associated with an increased risk of orthopaedic surgery compared with remission mean DAS28 (⩽2.6) [HR 2.59 (95% CI 1.49, 4.52)] [12].

These studies give us an insight into the association between factors early in disease progression and orthopaedic surgery, but there is little understanding of the association between time-varying factors and surgery. In the era of stratified medicine, pinpointing patients who are at high risk for surgery in the next year is perhaps more useful than identifying patients at moderate risk over the next 5–20 years [13]. Furthermore, although it has been shown that joint erosion is predicted by local inflammation [14, 15], it is unclear whether swelling, a marker of local inflammation, and tenderness in a specific joint at the early stages of the disease process predict later surgery on that joint. Lastly, observational research on outcomes after surgery in RA patients often focuses on negative outcomes and complications [16, 17], and there has been little research on whether surgery improves patient outcomes [18].

This study assesses the predictors of and outcomes following major orthopaedic surgery in an inception cohort of patients with early RA recruited by the Norfolk Arthritis Register (NOAR) and followed for 20 years. The primary objective of this study was to assess the relationship between baseline and time-varying clinical and demographic variables and both first and multiple major joint surgery. Additional objectives were to assess whether disease activity in a particular joint early after symptom onset predicts surgery on that joint and to assess whether functional disability improves following surgery.

## Methods

Patients in this study were recruited to the NOAR between 1990 and 1994. Detailed information on the NOAR can be found elsewhere [19]. In brief all incident cases of patients with inflammatory polyarthritis (IP) presenting at primary care in the former Norwich Health Authority region, Norfolk UK, were referred to the NOAR by primary care physicians or their consultant rheumatologists (*N* = 1098). The inclusion criteria of the NOAR were age ⩾16 years and two or more swollen joints lasting for ⩾4 weeks. Only patients recruited within 2 years following symptom onset were included in this analysis (76 patients were excluded). The 2010 ACR/EULAR RA criteria were applied retrospectively at baseline and only patients classified as RA according to these criteria were included in the main analysis (614 patients with RA were included) [20]. Lastly, patients were required to attend at least one follow-up assessment to report incident surgery; thus a total of 589 patients were included. Patients gave written informed consent in accordance with the Declaration of Helsinki and the NOAR was approved by the Norfolk and Norwich University Hospital Local Research Ethics Committee. This study, as an analysis of the data from the NOAR, did not require additional approval.

### Assessments

Patients were assessed at baseline and at years 1, 2, 3, 5, 7, 10, 15 and 20. Patients were only assessed beyond year 5 if they had documented swollen joints on two or more occasions or had received DMARDs or oral corticosteroids by the fifth-year assessment (seven patients were excluded after follow-up 5). Gender and date of birth were recorded at baseline. At each assessment patients reported start and stop dates for all prescribed DMARDs and corticosteroids as well as incident comorbidities, which were coded based on the International Classification of Diseases (revisions 9 and 10). A research nurse performed a 51 joint examination (other than years 5 and 7) and recorded each assessed joint as swollen, tender or both; swollen (SJC28 and 51) and tender (TJC28 and 51) joint counts were calculated. The 51 joints included in the assessment were neck, shoulders, elbows, wrists, MCP joints, PIP joints, DIP joints, hips, knees, ankles and MTP joints. Patients completed the British version of the HAQ Disability Index (HAQ-DI) [21]. Blood samples were taken at baseline and every 5 years, aliquoted and stored in freezers at − 80 °C. RF (latex test, positive cut-off 40 units/ml) and anti-CCP antibody (tested using the Diastat Anti-CCP2 kit, Axis-Shield, Dundee, UK; cut-off 5 units/ml) positivity were tested from baseline samples and CRP (mg/l) level at baseline and every 5 years. The three-component DAS28 (i.e. CRP, SJC28, TJC28) was calculated [22].

#### Surgery assessment

Patients reported on four facets of surgery: type, site, laterality and year. Only synovectomy, excision, fusion/arthrodesis and replacement surgeries performed on the shoulder, elbow, wrist, hip, knee or ankle were included in this analysis. These were grouped as either replacement or non-replacement surgery. Self-reported surgeries were validated by comparing patient-reported surgical interventions with those recorded in hospital notes. Agreement was good (κ > 0.90) between whether a surgery took place and the type, site and year of surgery and therefore self-reported surgeries were used as the outcome (for details see supplementary data section Surgery validation procedure and results, available at *Rheumatology* Online).

### Statistical analysis

Baseline characteristics, stratified by whether or not patients reported surgery [reported surgery (RS), no reported surgery (N-RS)], were described using descriptive statistics and compared using either chi-squared or Mann–Whitney U tests, depending on the type of data.

A Kaplan–Meier survival curve was plotted for the time to the first major surgery. The association between demographic and clinical variables and risk of first surgery was analysed using a Cox proportional hazards model; the proportional hazards assumption was met. Patients were censored at their last follow-up visit. Initially only baseline variables were used as predictors in a multivariate model: age, gender, HAQ score, SJC51, TJC51, anti-CCP2, RF, CRP and taking DMARDs. In a second model, baseline age, HAQ score, SJC51, TJC51, CRP and taking DMARDs were replaced by the equivalent time-varying variables. If a follow-up assessment did not assess a particular variable, then the value of that variable at the closest previous assessment where it was measured was used. Median age, SJC51, TJC51, HAQ and the number and proportion of patients taking synthetic DMARDs and biologic DMARDs at the start of each follow-up window were reported, stratified by whether a patient had surgery in the follow-up window. The scores were compared using chi-squared or Mann–Whitney U tests, depending on the type of data. A follow-up window was defined as the time between two attended follow-ups. Predictors of surgery (i.e. not just first surgery) were assessed using a conditional risk set model [23]. Each year was considered as a risk set. Each patient started in risk set 1, indicating no surgeries and after each surgery patients moved into the next risk set. Therefore the conditional risk set at a particular time for the *n*th surgery is made up of all follow-up time for patients who had *n* − 1 surgeries [24]. A Cox proportional hazards model was then used, stratifying by risk set. The same covariates were used as when analysing the first surgery. The analyses were then limited to lower limb (hip, knee, ankle) surgery and the HAQ was substituted with the walking and rising HAQ subcomponents in two separate models to assess the association between specific surgery sites and certain daily activities.

The relationship between early disease activity (swollen joint at baseline) in a particular joint and subsequent surgery on that joint was assessed using a Cox proportional hazards model. To assess whether early cumulative disease activity in a joint was associated with surgery on that joint, the number of assessments out of the first four at which the patient was recorded as having swelling in that joint was used as the independent variable. Follow-up time for this subanalysis began after the patient’s fourth assessment, meaning that a number of surgeries were left-censored (14–20%). This subanalysis was limited to the patients who attended baseline and follow-ups 1, 2 and 3 plus at least one other to report incident surgery. Due to the low number of surgical interventions for some joints, the subanalysis was only conducted on the knee joint and adjusted for age, gender and tenderness. The analysis was repeated with tender joints at baseline/over the first four assessments as the independent variable.

To analyse outcome after surgery, the change in HAQ score over each follow-up window was calculated. Then the changes in HAQ over follow-up windows where surgery was reported/not reported were compared using a mixed effects linear regression model, initially only controlling for age and gender. Then a weighted model was used controlling for gender, baseline RF and anti-CCP2, time-varying age, SJC51, TJC51, CRP, HAQ and comorbidities. As patients who required joint surgery were likely to have more severe disease and patients who leave the cohort are likely to be different from those who remain, the model was weighted using inverse probability of treatment/censoring weights [25, 26]. The analysis was then limited to lower limb surgeries and the walking and rising subcomponents of the HAQ to assess whether lower limb surgery was associated with improvements in these areas of daily living.

All regression analyses were repeated, confined to the total the NOAR IP population (*n* = 964) recruited from 1990 to 1994 and with at least one follow-up (see supplementary Tables S1–S3, available at *Rheumatology* Online, for these results). Multiple imputation using chained equations was performed to account for missing data of predictors. All analyses were performed using Stata 13.1 (StataCorp, College Station, TX, USA).

## Results

A total of 589 patients with RA were included in this analysis. The analysis included a total of 7185 years of follow-up (patient-years). All patients contributed at least 1 year of follow-up, up to a maximum of 20 [mean 12.1 years (s.d. 7.1)]; 260 (44.1%) patients died during follow-up, 116 (19.7%) patients declined further participation in the project or were lost to follow-up and 213 (36.2%) patients were assessed at year 20.

Over this time, 206 major surgeries were self-reported by 102 (17.3%) patients. Of these, 180 were included in the analysis, as 26 were duplicate reports. The median length of time between baseline and the first reported surgery of patients was 8 years [interquartile range (IQR) 4–12]. These 180 surgeries included 145 (80.5%) replacement surgeries and 35 (19.4%) non-replacement surgeries. The most commonly reported surgery was hip replacement surgery [*n* = 68 (37.7% of self-reported surgeries)] followed by knee replacement surgery [*n* = 58 (32.2%)] (see supplementary Table S4, available at *Rheumatology* Online, for frequencies of all reported surgeries). A similar proportion of patients underwent surgery in the total IP population [*n* = 151 (15.6%)].

### Baseline characteristics

The 589 included patients had a median age at baseline of 56 years (IQR 45–68) and 393 (66.7%) were women. Patients who had surgery and those who did not have surgery were of similar age at symptom onset [median age: RS = 57 years (IQR 47–66), N-RS = 55 years (IQR 43–68)] and included a similar proportion of women (66 *vs* 71%). The only differences between the groups at baseline were that patients who subsequently underwent surgery had lower TJC scores [median TJC28: RS = 7 (IQR 4–13), N-RS = 10 (IQR 5–17); median TJC51: RS = 11 (IQR 6–20), N-RS = 15 (IQR 7–23)]; higher CRP levels [median CRP: RS = 9.5 mg/l (IQR 4–36.3), N-RS = 7 (IQR 1–18)] and a higher proportion of anti-CCP2-positive patients [RS = 55.2% (*n* = 48), N-RS = 37.8% (*n* = 148); Table 1].

**Table 1:** Baseline characteristics of the cohort, stratified by whether they ever reported surgery

Characteristics	Total cohort		Reported surgery over follow-up		Did not report surgery over follow-up		
	*n*	Median (IQR)	*n*	Median (IQR)	*n*	Median (IQR)	P-value	
Age at onset, years	589	56 (45–68)	102	57 (47–66)	487	55 (43–68)	0.814^a^
Gender, female, *n* (%)	393 (66.7)		72 (70.6)		321 (65.9)		0.362^b^
Symptom duration before baseline assessment, months	589	5.2 (2.9–9.7)	102	4.6 (2.9–9.1)	487	5.4 (2.9–9.9)	0.461^a^
Swollen joints							
28	589	9 (4–14)	102	8 (5–12)	487	9 (4–15)	0.471^a^
51	589	11 (6–18)	102	11 (6–16)	487	11 (6–18)	0.834^a^
Tender joints							
28	589	10 (4–16)	102	7 (4–13)	487	10 (5–17)	0.024^a^
51	589	14 (7–23)	102	11 (6–20)	487	15 (7–23)	0.046^a^
CRP, mg/l	497	8 (2–21)	90	9.5 (4–36.3)	407	7 (1–18)	0.004^a^
DAS28	497	4.6 (3.8–5.6)	90	4.79 (4.01–5.40)	407	4.59 (3.82–5.63)	0.664^a^
HAQ	583	1.13 (0.50–1.63)	100	1.13 (0.50–1.75)	483	1.13 (0.50–1.63)	0.332^a^
Smoking status, *n* (%)	589		102		487		
Never	176 (29.9)		30 (29.4)		146 (30.0)		0.013^b^
Ever	252 (42.8)		55 (53.9)		197 (40.5)		
Current	161 (27.3)		17 (16.7)		144 (29.6)		
RF status, *n* (%)	536		96		440		
Positive	214 (39.9)		42 (43.8)		172 (39.1)		0.398^b^
Negative	322 (60.1)		54 (56.3)		268 (60.9)		
Anti-CCP2 status, *n* (%)	479		87		392		
Positive	196 (40.9)		48 (55.2)		148 (37.8)		0.003^b^
Negative	283 (59.1)		39 (44.8)		244 (62.2)		
Taking sDMARDs, *n* (%)	120 (20.4)		25 (24.5)		95 (19.5)		0.254^b^
Follow-up time, person-years	7185		1633		5552		

a Mann-Whitney U test.

b Chi-squared test. IQR: interquartile range.

### Predictors of first surgery

**Fig. 1:**
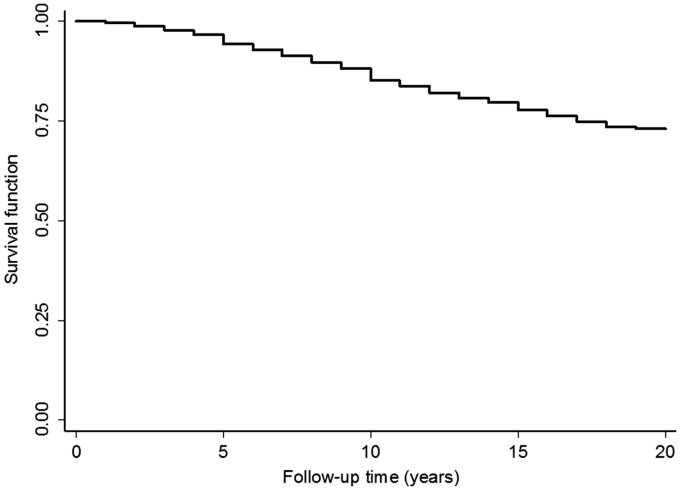
A Kaplan–Meier graph showing the time to the first major orthopaedic surgery

Figure 1 shows the Kaplan–Meier survival curve of time to first surgery. Age and anti-CCP2 positivity were the only baseline characteristics independently associated with an increased risk of first surgery [HR 1.02/year increase in age (95% CI 1.01, 1.04); HR 1.84 anti-CCP2 positive *vs* anti-CCP2 negative (95% CI 1.01, 3.38)]. HAQ scores at the start of a follow-up window were consistently higher in the patients who had a surgery within that follow-up window, whereas there were no differences in the SJC51, TJC51 and proportion receiving disease-modifying treatment between groups (Table 2). Using time-varying variables in the Cox regression, the HAQ score at the beginning of a follow-up window was independently associated with the risk of first surgery during that window [HR 2.19/unit increase in HAQ score (95% CI 1.66, 2.88)].

**Table 2:** Age, disease activity, disability and treatment over time, stratified by whether patients reported surgery

	0–1		1–2		2–3		3–5		5–7		7–10		10–15		15–20	
Variable	RS	N-RS	RS	N-RS	RS	N-RS	RS	N-RS	RS	N-RS	RS	N-RS	RS	N-RS	RS	N-RS
Age, median, years (*n*)	64 (4)	57 (585)	74 (3)	58* (546)	58 (6)	59 (514)	62.5 (20)	59 (451)	58 (19)	59 (398)	64 (27)	61 (338)	67 (30)	61 (251)	69 (22)	63* (189)
SJC51, median (*n*)	13.5 (4)	11 (585)	8 (3)	3 (543)	4.5 (6)	2 (499)	3.5 (20)	2 (427)	–	–	–	–	3 (30)	2* (249)	2 (22)	1 (189)
TJC51, median (n)	13 (4)	14 (585)	9 (3)	6 (543)	10.5 (6)	6 (499)	4.5 (20)	5 (427)	–	–	–	–	5.5 (30)	3 (249)	6 (22)	6 (189)
HAQ, median (*n*)	1.81 (4)	1.13 (579)	1.00 (3)	0.75 (545)	1.63 (6)	0.75 (511)	1.56 (20)	0.88** (446)	1.88 (19)	1.00** (398)	1.88 (27)	1.00* (330)	1.75 (30)	0.88** (247)	1.81 (22)	1.00** (189)
On sDMARD^a^, *n* (%)^b^	1 (25.0)	116 (19.8)	2 (66.7)	204 (37.4)	3 (50.0)	208 (40.5)	12 (60.0)	184 (40.8)	9 (47.4)	157 (39.5)	13 (48.2)	138 (40.8)	13 (43.3)	110 (43.8)	12 (54.6)	85 (45.0)
On bDMARD^a^, *n* (%)^b^	0 (0.0)	0 (0.0)	0 (0.0)	0 (0.0)	0 (0.0)	0 (0.0)	0 (0.0)	0 (0.0)	0 (0.0)	0 (0.0)	1 (3.7)	0 (0.0)	2 (6.7)	5 (2.0)	4 (18.2)	16 (8.5)

Swollen and tender joint counts not performed at follow-up 5 and 7.

a Synthetic DMARDs (sDMARDs) included gold, penacillamine, SSZ, HCQ, MTX, AZA and CYC. Biologic DMARDs (bDMARDs) included etanercept, infliximab, adalimumab and rituximab.

b DMARD exposure complete for each patient, therefore *n* is the same as age.

* *P* < 0.05,

** *P* < 0.005. RS: had surgery in follow-up window; N-RS: did not have surgery in follow-up window.

### Predictors of multiple surgeries

All 180 surgeries were included in the conditional risk set analysis. Baseline age [HR 1.01/year (95% CI 1.00, 1.03)] and anti-CCP2 positivity [HR 1.83 compared with anti-CCP2 negative (95% CI 1.07, 3.13)] were significantly associated with an increased risk of surgery during follow-up. The HAQ score at the start of a follow-up window independently predicted having surgery within that follow-up window [HR 2.11/unit increase in HAQ (95% CI 1.64, 2.71)].

### Walking and rising subcomponents of the HAQ

There was no significant association between the baseline score for the HAQ walking subcomponent [HR 0.85/unit increase (95% CI 0.63, 1.13)] or the rising subcomponent [HR 1.39/unit increase (95% CI 0.95, 2.06)] and the risk of lower limb surgery. However, there were significant associations between these scores at the start of a follow-up window and lower limb surgery within that window [HR 1.70/unit increase in walking subcomponent (95% CI 1.35, 2.14); HR 1.38/unit increase in rising subcomponent (95% CI 1.07, 1.78)].

### Disease activity in a particular joint and surgery on that joint

Having a swollen knee at baseline was associated with a doubling of the risk of requiring surgery on that knee over the follow-up (Table 3). Furthermore, the risk of knee surgery increased as the number of assessments increased at which patients were found to have swelling in that knee (Table 3). There was no association between baseline/cumulative tender knee joint and subsequent knee surgery. Similar results were seen when the analyses were confined to the whole IP population and to replacement surgeries (supplementary Table S5, available at *Rheumatology* Online).

**Table 3:** Baseline and cumulative knee joint disease activity association with subsequent knee joint surgery

	First left knee surgery	First right knee surgery
**Baseline**		
Number of surgeries included in the analysis	28	31
Time included in the analysis (years)	6998	6970
Swollen joint (compared with not swollen), HR (95% CI)	1.71 (0.71, 4.07)	2.54 (1.17, 5.50)
Tender joint (compared with not tender), HR (95% CI)	0.90 (0.39, 2.09)	0.91 (0.42, 1.98)
**Cumulative**		
Number of surgeries included in the analysis	24	28
Time included in the analysis (years)	5079	5073
Number of assessments out of first four where joint was swollen, HR (95% CI)		
0, [*n*]	[266] 1	[229] 1
1, [*n*]	[100] 3.04 (0.81, 11.46)	[122] 3.06 (0.91, 10.29)
2, [*n*]	[50] 9.65 (2.26, 41.18)	[57] 4.72 (1.30, 17.19)
3, [*n*]	[39] 8.87 (1.90, 41.34)	[39] 6.82 (1.62, 28.81)
4, [*n*]	[9] 16.29 (2.16, 122.52)	[17] 12.14 (2.17, 67.80)
Number of assessments out of first four where joint was tender, HR (95% CI)		
0, [*n*]	[190] 1	[177] 1
1, [*n*]	[120] 0.59 (0.16, 2.24)	[124] 5.82 (1.23, 27.56)
2, [*n*]	[73] 0.44 (0.10, 1.90)	[82] 2.87 (0.51, 16.32)
3, [*n*]	[47] 0.46 (0.10, 2.15)	[45] 3.91 (0.61, 25.02)
4, [*n*]	[34] 0.45 (0.09, 2.26)	[36] 2.00 (0.27, 14.92)

Covariates included in baseline models: age at onset, gender and swollen joint count and tender joint count at baseline. Covariates included in cumulative models: age at onset, gender and number of assessments out of the first four where the patient had a swollen joint and the number that had tender joints on the outcome joint. HR: hazard ratio.

### Outcome after surgery

Patients who had a surgery in a follow-up window had greater deterioration in the HAQ score over that follow-up window than patients who did not have a surgery, in an age- and gender-adjusted model (Table 4). After weighting the model to attempt to account for confounding by indication, patients who required surgery still had a greater deterioration in HAQ scores over follow-up windows compared with patients who did not have surgery. Similar results were seen when limiting the analysis to walking and rising subscales of the HAQ and lower limb surgery.

**Table 4:** Change in HAQ scores over follow-up windows; reported surgery *vs* no reported surgery

Comparison	RA patients – adjusted for age, gender and HAQ at the start of follow-up window, β (95% CI)	RA patients – further adjusted,^a^ β (95% CI)	Total IP cohort – adjusted for age, gender and HAQ at the start of the follow-up window, β (95% CI)	Total IP cohort – further adjusted,^a^ β (95% CI)
Change in HAQ score over the follow-up window				
Did not have surgery	0	0	0	0
Had surgery	0.18 (0.09, 0.27)	0.17 (0.03, 0.32)	0.17 (0.11, 0.24)	0.20 (0.08, 0.33)
Did not have replacement surgery	0	0	0	0
Had replacement surgery	0.16 (0.06, 0.26)	0.14 (−0.01, 0.29)	0.16 (0.08, 0.24)	0.20 (0.07, 0.34)
Change in walking subcomponent over the follow-up				
Did not have lower limb surgery	0	0	0	0
Had lower limb surgery^b^	0.27 (0.13, 0.41)	0.27 (0.06, 0.48)	0.28 (0.17, 0.38)	0.27 (0.07, 0.47)
Did not have lower limb replacement surgery	0	0	0	0
Had lower limb replacement surgery^b^	0.23 (0.09, 0.37)	0.21 (−0.01, 0.42)	0.18 (0.07, 0.29)	0.19 (−0.01, 0.40)
Change in rising subcomponent over the follow-up				
Did not have lower limb surgery	0	0	0	0
Had lower limb surgery^b^	0.23 (0.10, 0.36)	0.17 (−0.07, 0.41)	0.19 (0.09, 0.30)	0.30 (0.10, 0.49)
Did not have lower limb replacement surgery	0	0	0	0
Had lower limb replacement surgery^b^	0.16 (0.02, 0.29)	0.13 (−0.10, 0.36)	0.19 (0.09, 0.30)	0.30 (0.10, 0.49)

a Controlling for baseline anti-CCP2, gender, 2010 RA status and RF; time-varying: age, CRP, SJC51, TJC51, comorbidities and HAQ score at the start of a follow-up window.

b Lower limb surgeries included hip, knee or ankle surgery.

## Discussion

Within the NOAR cohort, 17.3% of RA patients required at least one major orthopaedic surgery over the course of 20 years and therefore it would appear the need for orthopaedic surgery has fallen in recent years. A study of 1600 prevalent RA patients recruited in 1974 in the USA found that 33.8% required any orthopaedic surgery and 17.8% of patients required total joint replacement over 23 years [27]. A study following 112 RA patients from 1964 to 1986 in Droitwich, UK reported 26.8% of patients required reconstructive surgery [28].

Despite the reduction in frequency of orthopaedic surgery in the modern era, time-varying HAQ scores were strongly associated with an increased risk of surgery in both the 1974 US study and within this analysis [27]. An association between HAQ score and subsequent orthopaedic surgery was also reported from the Lund early RA cohort [10] and the Utrecht Rheumatoid Arthritis Cohort [29]. HAQ score has also been shown to predict premature mortality [30] and work disability [31, 32] in patients with IP, emphasizing the value of regular measurement of the HAQ in routine clinical practice.

Furthermore, this analysis demonstrated a strong association between knee swelling and subsequent surgery of that knee. Similar results were reported from a Japanese cohort of 1134 prevalent cases of RA followed for 5 years, with total knee replacement as the outcome [knee involvement at baseline *vs* no knee involvement: HR 4.01 (95% CI 2.46, 6.52)] [33]. However, Yasui et al*.* [33] combined left and right knee activity and used this to predict later knee arthroplasty. Our analysis suggests that, to reduce the need for joint surgery, it may be important to target swelling in individual joints and not to focus solely on lowering the total SJC.

Lastly, this analysis showed that HAQ scores increased in patients who had surgery compared with patients who did not, even after attempting to control for confounding by indication. This could be because joint surgery is associated with reduced function in that joint or it could be that function improves in the operated joint but continues to deteriorate in non-operated joints. In other words patients who require joint surgery may have generally worse disease and operating on a single joint may have little impact on function in general.

Furthermore, if all patients who would benefit from surgery undergo surgery, there may be no overlap between the surgery and the non-surgery groups in terms of their combination of confounders, meaning that any attempt to balance the covariates using weighting is likely to be ineffective [26]. Lastly, the time between having surgery and the first post-operative HAQ score varied up to 5 years or more. However, restricting the analysis to only include the first 10 years of follow-up from baseline, and thus limit the potential time between surgery and assessment, produced similar results (results not shown).

This study had a number of strengths. This was a large inception cohort with little selection bias, including data on many demographic and clinical variables at baseline and during follow-up, and up to 20 years of follow-up. A potential weakness of this study is the reliance on patients self-reporting surgeries; however, these self-reports were validated by checking hospital records, with good agreement. Another weakness is that we do not know whether the joint replacements were carried out for the RA-related damage or primary/secondary OA. Unfortunately BMI was not measured at baseline in this group of patients and so we could not explore whether this would have been an independent predictor of future joint replacement. Likewise, DAS28 scores over the first 5 years of follow-up could not be assessed, as blood samples for CRP measurement were not taken at these time points [12].

In conclusion, we have shown that 17.3% of RA patients in 1990–94 underwent at least one major joint surgery in the 20 years following presentation. Hip replacement was the most common intervention, followed by knee replacement. The strongest predictor of joint surgery in both the short and long-term was the HAQ score. Regular measurement of the HAQ in routine clinical practice may help identify those who should be considered for surgery. However, joint surgery was not associated with an improvement in HAQ score in the medium to long term. This suggests that focusing on disease control in order to reduce the progression of joint damage and need for joint surgery is the best treatment strategy.

## Supplementary Material

Supplementary Tables
