# Supplementary material for: Predictors of and outcomes following orthopaedic joint surgery in patients with early rheumatoid arthritis followed for 20 years
Source: Rheumatology (Oxford). 2017 May 16;56(9):1510–7. doi: 10.1093/rheumatology/kex172 (PMC5850659; doi:10.1093/rheumatology/kex172)
Supplement: Supplementary Tables [file rhe-17-0045-file003_kex172.docx]

**SUPPLEMENTARY DATA**

**Surgery validation procedure and results**

This supplementary file contains the methods and results of the surgery validation that was carried out to ensure that the self-reported surgery data used in the analysis were valid.

**Method**

The self-reported surgeries were validated to ensure accuracy. A medical auditor at Norfolk and Norwich University Hospital screened electronic records of all NOAR patients who had reported surgeries, producing a database detailing admission and discharge dates, and procedure codes with text descriptions. Furthermore, a random 10% of patients who didn’t self-report a surgery also had their medical records screened to check for under-reporting.

In 2006 the medical records of patients who self-reported orthopaedic procedures up to that time were reviewed and a database containing information of operation type, site, laterality and year was created. This database was checked if a self-reported surgery was not validated by the auditor’s database and took place in or before 2006. A further hospital record screening of all patients who had surgery after 2006 and whose self-reported procedure had not been validated by the auditor’s database was carried out in 2016.

Finally, patients with un-validated self-reported surgeries had their NOAR paper questionnaires screened. On each assessment, the research nurse completed a manikin diagram marking operated joints. This method only allows validation of the site and laterality of an operation.

*Analysis*

The percentage agreement between the validation methods and the self-reports were calculated for whether a surgery took place and then for each of the four facets of the self-report. Kappa statistics were calculated which account for agreement that occurs by chance [1]. Kappa statistics between 0.61-0.80 are considered as substantial agreement and between 0.81-1 as almost perfect agreement [2].

**Results**

Over follow-up, 149 patients with IP reported having one or more surgeries (range 1 – 9) and 308 surgeries were self-reported in total. Of these, 257 were matched to a surgery recorded in one of the three validation methods, 35 were duplicate reports and 16 remained unmatched. A further 23 surgeries were found by the validation methods to have occurred that were not reported, but all 23 came from patients who had already reported another surgery; none of the random 10% of patients who did not report having a surgery were found to have a surgery recorded in their medical notes. Therefore there was 91.4% agreement on whether a surgery had taken place, with a kappa statistic of 0.78 (p<0.0001) indicating substantial agreement. Of these 257 surgeries, there were high levels of correct self-reporting on each of the four aspects: operation type = 98.1% agreement (kappa = 0.94, p<0.0001), operation site = 99.6% agreement (kappa = 0.99, p<0.0001), laterality of operation = 94.4% agreement (kappa = 0.89, p<0.0001), year of surgery = 88.2% (kappa = 0.88, p<0.0001).

**Discussion**

There was a good level of agreement between patients reporting whether a surgery took place and a surgery being listed in the hospital records for that patient. The reasons for discrepancies could be that some patients may have had operations in private care, or may have miss-reported the type of surgery they had (i.e. reporting a surgery that was included in our analysis, whereas they actually underwent a surgery that was not included in our analysis). Of the surgeries that were validated as having taken place, again there was good agreement on the four facets of the surgery. Patients almost perfectly reported on the type and operation site, and there was very high agreement on laterality. The year of the surgery was slightly less well reported, but as patients potentially could be reporting their surgery to the research team many years after the surgery took place it is unsurprising that some were reported incorrectly. Lastly 23 surgeries were found by the validation procedure to have occurred that were not reported. These all came from patients who had already reported another surgery. It is encouraging that none of the random 10% of patients who did not report a surgery were found to have a surgery in their hospital notes as it means that we can be confident that all patients who did not report having a surgery did not have a surgery and thus we don’t need to screen the whole cohort’s notes. In conclusion, this surgery validation procedure gave us confidence to use the self-reported surgery data, as it appears that patients report well on the surgeries that they have undergone.

**REFERENCES**

1. Viera AJ, Garrett JM. Understanding interobserver agreement: the kappa statistic. Fam Med 2005;37:360-3.

2. Landis JR, Koch GG. The measurement of observer agreement for categorical data. Biometrics 1977;33:159-74.

**Supplementary Table S1. Baseline characteristics of the total cohort, stratified by ever reporting surgery**

|  | Total Cohort | | Reported surgery over follow-up | | Did not report surgery over follow-up | |  |
| --- | --- | --- | --- | --- | --- | --- | --- |
|  | N | Median (IQR) | N | Median (IQR) | N | Median (IQR) | p |
| Age at onset, years | 964 | 54 (42–67) | 151 | 57 (47–66) | 813 | 53 (41–67) | 0.067^a^ |
| Gender, female, N (%) | 635 (65.9) |  | 103 (68.2) |  | 532 (65.4) |  | 0.509^b^ |
| Symptom duration before baseline assessment, months | 964 | 5.0 (2.7–9.3) | 151 | 4.5 (2.7–8.9) | 813 | 5.0 (2.6–9.3) | 0.497^a^ |
| Swollen joints:  28  51 | 964  964 | 5 (2–11)  6 (2–13) | 151  151 | 5 (2–11)  7 (3, 13) | 813  813 | 5 (1–11)  6 (2–13) | 0.236^a^  0.094^a^ |
| Tender joints:  28  51 | 964  964 | 5 (2–12)  7 (3–17) | 151  151 | 5 (2–10)  7 (3–15) | 813  813 | 5 (2–12)  7 (2–17) | 0.939^a^  0.888^a^ |
| CRP, mg/l | 779 | 5 (0–15) | 127 | 8 (2–29) | 652 | 5 (0–14) | 0.0001^a^ |
| DAS28 | 779 | 4.0 (2.9–5.1) | 127 | 4.3 (3.4–5.1) | 652 | 3.9 (2.8–5.0) | 0.017^a^ |
| HAQ | 952 | 0.75 (0.25–1.38) | 147 | 0.88 (0.38–1.63) | 805 | 0.75 (0.25–1.38) | 0.015^a^ |
| Smoke status  Never, N (%)  Ever, N (%)  Current, N (%) | 963  307 (31.9)  402 (41.7)  254 (26.4) |  | 151  47 (31.1)  79 (52.3)  25 (16.6) |  | 812  260 (32.0)  323 (39.8)  229 (28.2) |  | 0.003^b^ |
| RF status:  Positive, N (%)  Negative, N (%) | 847  239 (28.2)  608 (71.8) |  | 139  47 (33.8)  92 (66.2) |  | 708  192 (27.1)  516 (72.9) |  | 0.109^b^ |
| Anti-CCP status:  Positive, N (%)  Negative, N (%) | 756  208 (27.5)  548 (72.5) |  | 122  51 (41.8)  71 (58.2) |  | 634  157 (27.8)  477 (75.2) |  | <0.001^b^ |
| Taking sDMARDs, N(%) | 148 (15.4) |  | 35 (23.2) |  | 113 (13.9) |  | 0.004^b^ |
| Met 2010 RA criteria, N (%) | 589 (61.1) |  | 102 (67.6) |  | 487 (59.9) |  | 0.077^b^ |
| Follow-up time, person years | 11,706 |  | 2440 |  | 9266 |  |  |

^a^Mann-Whitney ;, ^b^ Chi squared χ^2^. Anti-CCP: Anti-citrullinated peptide antibody; N: Number of patients with available data.

**Supplementary Table S2. Regression analyses performed on the total IP cohort (N = 964)**

| Analysis | Total cohort all surgeries, HR (95% CI) | Analysis | Total cohort all surgeries, HR (95% CI) |
| --- | --- | --- | --- |
| *Association between baseline variables and risk of first surgery* |  | ***Association between time-varying variables and risk of surgery*** |  |
| Age | HR per year increase: 1.02 (1.00, 1.03) | HAQ | HR per unit increase: 2.15 (1.74, 2.65) |
| Anti-CCP2 positive | HR vs. Anti-CCP2 negative: 1.67 (0.97, 2.88) | CRP | HR per mg/l increase: 1.00 (1.00, 1.01) |
| *Association between time-varying variables and risk of first surgery* |  | ***Association between baseline subcomponents of the HAQ and lower limb surgery*** |  |
| HAQ | HR per unit increase: 2.30 (1.83, 2.90) | Walking | HR per unit increase: 1.05 (0.81, 1.36) |
| Age | HR per year increase: 1.02 (1.01, 1.03) | Rising | HR per unit increase: 1.32 (0.97, 1.78) |
| CRP | HR per mg/l increase: 1.00 (1.00, 1.00) |  |  |
| *Association between baseline variables and risk of surgery* |  | ***Association between time-varying subcomponents of the HAQ and lower limb surgery*** |  |
| Age | HR per year increase: 1.02 (1.00, 1.03) | Walking | HR per unit increase: 1.82 (1.48, 2.24) |
| Anti-CCP positive | HR vs. Anti-CCP2 negative: 1.52 (0.98, 2.35) | Rising | HR per unit increase: 1.33 (1.07, 1.65) |

**Supplementary Table S3. Baseline and cumulative knee joint disease activity’s association with subsequent knee joint surgery**

| *Baseline* | First left knee surgery | First right knee surgery |
| --- | --- | --- |
| Number of surgeries included in analysis | 39 | 43 |
| Time included in analysis (years) | 11975 | 11935 |
| Swollen joint (compared to not swollen), HR (95%CI) | 2.54 (1.24 , 5.23) | 2.11 (1.07, 4.15) |
| Tender joint (compared to not tender),  HR (95%CI) | 0.77 (0.36, 1.65) | 1.36 (0.69, 2.67) |
| *Cumulative disease activity over first 3 years* | | |
| Number of surgeries included in analysis | 31 | 37 |
| Time included in analysis (years) | 8322 | 8292 |
| Number of assessments out of first 4 swollen joint, HR (95%CI) |  |  |
| 0, [n] | [468] 1 | [434] 1 |
| 1, [n] | [150] 3.38 (1.20, 9.52) | [163] 3.55 (1.26, 10.06) |
| 2, [n] | [71] 6.85 (2.04, 22.97) | [78] 5.69 (1.86, 17.39) |
| 3, [n] | [51] 10.88 (3.13, 37.85) | [60] 5.97 (1.67, 21.31) |
| 4, [n] | [15] 16.66 (3.38, 82.12) | [20] 16.34 (3.70, 72.11) |
| Number of assessments out of first 4 tender joint, HR (95%CI) |  |  |
| 0, [n] | [383] 1 | [366] 1 |
| 1, [n] | [175] 0.95 (0.35, 2.54) | [180] 3.40 (1.15, 10.05) |
| 2, [n] | [94] 0.50 (0.14, 1.77) | [108] 2.40 (0.71, 8.06) |
| 3, [n] | [61] 0.64 (0.17, 2.39) | [60] 2.52 (0.64, 9.97) |
| 4, [n] | [42] 0.87 (0.24, 3.11) | [41] 1.75 (0.39, 7.97) |

Covariates included in baseline models: age at onset, gender and swollen joint at baseline and tender joint at baseline on the outcome joint. Covariates included in cumulative models: age at onset, gender and number of assessments out of the first four that a patient had swollen joint and the number that had tender joint on the outcome joint.

**Supplementary table S4. Frequency of self-reported surgeries at different sites by patients with RA**

| Site | Replacement | Not replacement | Total |
| --- | --- | --- | --- |
| Shoulder | 10 | 3 | 13 |
| Elbow | 4 | 2 | 6 |
| Wrist | 5 | 20 | 25 |
| Hip | 68 | 0 | 68 |
| Knee | 58 | 5 | 63 |
| Ankle | 0 | 5 | 5 |
| Total | 145 | 35 | 180 |

*A*

**Supplementary Table S5. Baseline and cumulative knee joint disease activity’s association with subsequent knee joint replacement surgery**

| *Baseline* | First left knee replacement surgery | First right knee replacement surgery |
| --- | --- | --- |
| Number of surgeries included in analysis | 26 | 28 |
| Time included in analysis, years | 7001 | 7350 |
| Swollen joint (compared to not swollen), HR (95%CI) | 2.07 (0.85, 5.04) | 2.71 (1.21, 6.07) |
| Tender joint (compared to not tender),  HR (95%CI) | 0.81 (0.34, 1.95) | 0.94 (0.42, 2.12) |
| *Cumulative disease activity over first 3 years* | | |
| Number of surgeries included in analysis | 22 | 29 |
| Time included in analysis (years) | 6941 | 6933 |
| Number of assessments out of first 4 swollen joint, HR (95%CI) |  |  |
| 0, [n] | [266] 1 | [229] 1 |
| 1, [n] | [100] 3.62 (0.89, 14.70) | [122] 2.68 (0.81, 8.91) |
| 2, [n] | [50] 9.88 (2.11, 46.36) | [57] 3.88 (1.09, 13.75) |
| 3, [n] | [39] 11.07 (2.21, 55.39) | [39] 3.89 (0.87, 17.31) |
| 4, [n] | [9] 19.98 (2.51, 159.15) | [17] 5.59 (0.84, 37.11) |
| Number of assessments out of first 4 tender joint, HR (95%CI) |  |  |
| 0, [n] | [190] 1 | [177] 1 |
| 1, [n] | [120] 0.79 (0.20, 3.13) | [124] 12.92 (1.61, 103.67) |
| 2, [n] | [73] 0.62 (0.13, 2.87) | [82] 5.67 (0.59, 54.30) |
| 3, [n] | [47] 0.71 (0.14, 3.49) | [45] 11.63 (1.15, 117.13) |
| 4, [n] | [34] 0.90 (0.19, 4.39) | [36] 6.49 (0.57, 74.14) |

Covariates included in baseline models: age at onset, gender and swollen joint at baseline and tender joint at baseline on the outcome joint. Covariates included in cumulative models: age at onset, gender and number of assessments out of the first four that a patient had swollen joint and the number that had tender joint on the outcome joint.
